# Bibliography

**Published:** 1903-04-15

**Authors:** 


### BIBLIOGRAPHY.

A NEW text-book on Dental Materia Medica and Therapeutics has just come from the pen of Eli H. Long, M.D., and the press of Lea Brothers & Co., of Philadelphia. Dr. Long is professor of Medical and Dental Materia Medica and Therapeutics in the University of Buffalo. The book he has written shows a remarkable familiarity with the needs of the dental student for a medical man. It contains a very good classification of remedies from their physiologic and therapeutic bearings as used in dental practice. He has either had some good coaching or some dental experience to enable him to compile a work so well adapted for the purpose for which it is made. No one has yet written a book which meets every need and which could pass without some criticism, and this work is remarkable from the fact that it furnishes so little opportunity for adverse comment. So valuable a class of therapeutic remedies as local stimulants get all too scant notice, and no adequate discrimination seems to be made between cauterants and escharotes. The subject of antiseptics is not sufficiently comprehensive or detailed to cover this important branch of dental therapeutics. The subject of bleaching agents is very brief indeed, but rather satisfactory. The subject of anesthetics, both local and general, is very well done, as are also the systemic remedies which come within the sphere of the dental therapeutist. Several valuable and well-designed drawings showing the pharmacologic action of various systemic remedies add materially to the text in clearly setting forth drug actions on the nervous system and vital organs. An index of drugs, giving the reaction, solubility, dose and use of every authoritative drug in use, add much to the value of the book as a reference work. We congratulate Dr. Long on his ability to make a text-book at once complete, compact and sufficiently inclusive to make it one of the best works of its kind extant for class purposes.

